# Small area analysis of HIV viral load suppression patterns in a high priority district (2012–2016), South Africa

**DOI:** 10.1371/journal.pgph.0001728

**Published:** 2023-03-31

**Authors:** Lucy Chimoyi, Tendesayi Kufa, Zvifadzo Matsena-Zingoni, Florian Marx, Kennedy Otwombe, Eustasius Musenge, Salome Charalambous

**Affiliations:** 1 Implementation Research Division, The Aurum Institute, Johannesburg, South Africa; 2 School of Public Health, University of the Witwatersrand, Johannesburg, South Africa; 3 Centre for HIV & STIs, National Institute for Communicable Diseases, National Health Laboratory Services, Johannesburg, South Africa; 4 DSI-NRF South African Centre of Excellence in Epidemiological Modelling and Analysis, Faculty of Science, Stellenbosch University, Stellenbosch, South Africa; 5 Desmond Tutu TB Centre, Department of Paediatrics and Child Health, Faculty of Medicine and Health Sciences, Stellenbosch University, Cape Town, South Africa; 6 Perinatal HIV Research Unit, Faculty of Health Sciences, University of the Witwatersrand, Johannesburg, South Africa; University of Embu, KENYA

## Abstract

Globally, high viral load (VL) suppression rates are indicators of successful HIV treatment programs. Evaluation of these programmes at lower levels is likely to highlight variations that are masked at the provincial or national levels. This ecological study used routinely collected clinical and surveillance data on the HIV programme from 88 sampled Ekurhuleni wards. Between January 2012 and December 2016, 26 222 HIV VL tests for 2817 patients were conducted. We conducted a secondary analysis to determine the predictors of high VL suppression accounting for space and time random effects and estimate the impact of the national universal test-and-treat roll-out in 2016 and forecast VL suppression rates for five years post-2016.The proportion of VL suppression increased over the years: 2012 (47.8%: 95% confidence interval (CI): 36.7%-67.4%); 2013 (58.2%: 95%CI: 41.4%-79.6%); 2014 (62.7%: 95%CI: 45.2%-84.7%); 2015 (67.2%: 95%CI: 49.0%-89.9%) and 2016 (61.2%: 95%CI: 43.9%-83.0%). For every percentage increase in ART initiation, high VL suppression rates increased by 35% (RR: 1.345; 95% credible interval (Crl) 1.221–1.492) and for every percentage increase in women in the ward, high VL suppression increased by 44% (RR: 1.442; 95%CrI: 1.056–1.962). There was evidence of high and low clusters of viral load suppression observed at ward-level. The VL suppression rates in Ekurhuleni were lower than the 90% UNAIDS target. There was heterogeneity of high VL suppression across wards and study period. Targeted interventions strengthening ART initiation and retention in care are critical to achieving optimal VL suppression in Ekurhuleni and districts with similar profiles.

## Introduction

Evaluating the effectiveness of HIV treatment programmes in a population through viral load (VL) monitoring is widely used in many HIV high-burden countries [1, 2]. There has been massive ART roll-out and scale-up of viral load monitoring since 2013 to meet the previous (90-90-90) and current (95-95-95) UNAIDS targets by 2030 and achieve ultimate HIV control [1]. HIV control can be attained when people living with HIV (PLHIV) are identified, initiated on ART, and retained in HIV care [2]. Therefore, high ART coverage and high rates of viral load suppression are likely to reduce onward HIV transmission and reduce HIV prevalence [1, 3–6].

In South Africa, HIV treatment initiation is done primarily at primary health care (PHC) facilities and viral load testing is initially performed bi-annually (at 6 and 12 months), and thereafter annually if suppressed [7]. If the viral load is greater than 1000 copies/mL, repeat testing is required according to World Health Organization (WHO) guidelines [6]. The 2020 UNAIDS data for South Africa shows that 92% of people were aware of their HIV status, 72% were on treatment and 66% were virally suppressed [1]. Since tangible evidence has demonstrated the significant impact that treatment has on the reduction of HIV transmission at a population level, population viral load is commonly monitored in resource-constrained settings [1, 6]. Heterogeneity of HIV prevalence has been widely demonstrated [8–12]. Moreover, viral load suppression rates are likely to vary spatially. To curb HIV transmission, there is a need to find alternative ways of monitoring the viral load while considering heterogeneity across geographical spaces and time [13]. Although viral load suppression in South Africa is reportedly 66%, there is a likelihood of variation at a sub-national level [1].

Locally, little is known about the estimates of viral load suppression and their association with related health outcomes. This limited information may be attributed to the complex interplay of risk factors that remain inadequately characterised in the spatial and temporal domain. Individual viral load suppression predictors in adults identified in previous research are female sex, older age, and early and timely ART initiation [14, 15]. However, an improved understanding of population-level viral suppression and its extent to reducing HIV incidence is needed. Given the variety of these individual predictors, addressing heterogeneities in viral load suppression by using predictive models could facilitate targeted prevention interventions while focusing on the delivery of these interventions [16].

To augment findings from predictive modelling, forecasting through the time-series approach can be used for particularly short-term time series prediction. The autoregressive integrated moving average (ARIMA) model is useful in evaluating and creating a forecasting model by modelling correlations in the datasets and has been used in non-healthcare sectors. In the healthcare sector, the model has been used in forecasting diseases such as future incident malaria cases and deaths in Ethiopia [17], and the prevalence of opportunistic infections in HIV patients in Uganda [18]. ARIMA models could be used to forecast trends with reasonable accuracy which provide early warnings for the future so that policy planners can appropriately allocate resources for interventions at the facility or ward level.

Studies utilizing the Bayesian techniques in mapping health outcomes are increasing as they leverage information on the disease events across neighbouring geographical units and the prior distribution of health outcomes. Small area estimation using Bayesian methods overcomes this problem by integrating prior information to the underlying relative risk, making the predicted risk estimates robust [19]. The statistical methodology for using Bayesian models for surveillance in space-time is still work in progress, but it is an attractive tool that allows the local understanding of disease processes integrated via the specification of prior distributions on model parameters.

Whereas Bayesian models have been extensively used in disease mapping studies, studies mapping viral load suppression in South Africa are limited. We used five years of HIV program data for Ekurhuleni, South Africa to assess the trends in viral load suppression rates, explored spatio-temporal autocorrelation at a ward level, and the immediate impact of universal test and treat (UTT) roll-out in 2016. Lastly, we developed a model from January 2012 to December 2016 and forecast viral load suppression from January 2017 to December 2021.

## Methods

### Study setting

This ecological study utilized aggregated data on HIV indicators collected from PHC facilities in Ekurhuleni Metropolitan Municipality (EMM) between 2012 and 2016. EMM is divided into three sub-districts (East, South and North) and 101 wards. Available spatial data was recorded for 88 wards. Most wards have one PHC.

### Data sources

Data on HIV viral loads were obtained from the South Africa National Health Laboratory Services (NHLS). NHLS conducts all laboratory monitoring for the national HIV program which is archived at the level of the laboratory specimen in the NHLS corporate data warehouse (CDW). Longitudinal data between January 2012 and December 2016 from Ekurhuleni with information on gender, age, tuberculosis (TB) diagnosis, ART start date and viral load count records were used.

Data on ART uptake at the clinic level in Ekurhuleni was abstracted from the District Health Information System (DHIS) hosted by the South Africa National Department of Health, which routinely collects individual-level data from all South African public health facilities at a local level while data on literacy levels, income levels, gender and population were obtained from the 2011 National Census. The combined anonymized dataset is provided in S1 Data.

### Viral load measure

The South African National Guidelines state that routine viral load monitoring for individuals receiving ART should be done at 6 and 12 months after ART initiation and then annually thereafter if VL is suppressed [7]. During each routine visit, a standard request laboratory form containing patient identifiers is manually captured into the laboratory information system at the receiving laboratory, before the blood sample is centrifuged and evaluated using the Abbott Realtime HIV (Abbott Molecular des Plaines, USA) or Roche Cobas Ampliprep/Cobas TaqMan HIV-1 (Roche Diagnostics, Branchburg, USA) assays. All viral load results from each patient during the study time period were used. The total number of patients with a viral load test during the time period was calculated.

### Viral load suppression (VLS) measures

For both outcomes of viral load suppression (VLS) (i) <400copies/mL (SA guidelines [7]) and (ii) <1000 copies/mL (WHO guidelines [6])), anonymized data from patients who accessed HIV care and had at least recorded a clinic visit during the study period, were used. However, this analysis considered <1000 copies/mL as the main outcome. We summarized the mean log_10_ viral load per year.

### Ward-level measures

We estimated the population characteristics at the ward level from 2011 Census data. Eight-eight wards were identified using geographical information provided in the 2011 Census data. We calculated the following measures for each ward: proportion of female respondents; the proportion of low literacy levels; the proportion with no income; and population density per ward from the 2011 Census data and performed standardization. We further interpolated subsequent estimates from midyear population estimates produced by Statistics South Africa from 2012–2016 [20] assuming that the population is growing at a constant rate using the exponential growth model. ART initiation rate per ward was calculated by estimating the total HIV patients in each ward remaining on ART at end of the month over the estimated number of people living with HIV. An arithmetic mean by ward was calculated for each year. Ward-level VLS was estimated by taking the number of virally suppressed patients and dividing it by the total number in care per year for each ward. As the per the UNAIDS minimum threshold, wards with ≥ 75% of PLHIV reporting VLS were considered having high VLS whereas wards with <75% were considered as having low VLS [2]. We further used this cut-off to generate a dichotomous variable (high VLS = 1 and low VLS = 0).

### Statistical analyses

#### Five-year trend analysis

We summarized the overall proportion of viral suppression by frequencies and percentages at the municipality level and sub-district level using tables and line graphs. To assess for the trend of viral load suppression and ART initiation rate across the five years, we used the Cochran-Mantel-Haenszel stratified test of association and Kruskal-Wallis equality of populations. All statistical analyses were performed in Stata version 16.1 [21]. *P*-values <0.05 were considered statistically significant.

#### Interrupted time series regression on viral load suppression in 2016

Two periods were specified: The preintervention (January–August 2016)—8 months before the roll-out of UTT, excluding September 2016 when UTT was rolled out nationally; and the postintervention (September 2016–December 2016)—4 months of UTT implementation.

#### Forecasting viral load suppression post 2016

We collapsed the VLS monthly data into quarters and smoothed out the series using a centred moving average to understand the underlying growth component. For forecasting accuracy, we divided the data into two groups: first, one for model development (January 2012 to December 2016) and the other for model validation (January 2017 to December 2021). Using the auto-regressive integrated moving average (ARIMA), we modelled the time series and applied it to VLS in Ekurhuleni.

#### Spatio-temporal Bayesian analysis

We examined the predictors of the proportion of viral load suppression at the ward level by fitting spatial and spatio-temporal mixed-effects regression model using the R Integrated Nested Laplace Approximation (INLA) package in R version V.3.6.1 (RStudio). The mixed-effects model accounted for viral load suppression as fixed effects with ward-specific random intercepts to account for overdispersion or correlation in viral load suppression within and between wards over five years. The adjusted viral load suppression rates were mapped using ArcGIS version 10.7.1. To assess spatial autocorrelation of viral load suppression in Ekurhuleni, we used the Moran index and to map clusters of high and low viral load suppression, we used the Anselin Local Indicator Spatial Autocorrelation (LISA) function in ArcGIS.

#### Spatial-temporal regression analysis

We also used the spatial statistics function in ArcGIS (Environmental Systems Research Institute, Redlands, USA) to i) explain the local relationship between high VLS and the two covariates (female population, and ART initiation) using ordinary least squares regression (OLS), ii) show how the two covariates changed from one year to another using geographically weighted regression (GWR).

### Sensitivity analysis

We conducted a sensitivity analysis by investigating the predictors of viral load suppression using <400copies/mL as an additional outcome. In addition, we restricted the analysis to include all observations until August 2016 before the national roll-out of universal test and treat (UTT) began.

### Ethical considerations

Ethical clearance was sought from the Wits Human Research Ethics committee (HREC; M181088). Additional permission was sought and approved from the National and Gauteng Department of Health (to access DHIS data), NHLS ethics committee and the Ekurhuleni Metropolitan Municipality Research Committee. Due to the use of retrospective anonymized data, this study did not seek individual patient consent. Participant consent was specifically waived by the research ethics committee that approved this study.

## Results

### Participants’ characteristics

From January 2012 to December 2016, 41 644 samples were collected from mostly male patients (n = 25 631; 61.5%) and of median age of 39 (IQR: 14) years who visited 99 PHCs in 88 wards in EMM for their routine HIV care. From these, 26 222 HIV viral load samples from 2 817 patients were recorded. The differences across the calendar years were significant (p-value<0.05). Table 1 shows a summary of selected patient characteristics of the laboratory data by year.

**Table 1:** Characteristics of samples collected from primary health care facilities in Ekurhuleni (2012–2016).

Indicator	2012	2013	2014	2015	2016	Trend test p-value
Number of patients	1001	1371	1871	2125	2296	0.001
Median age (IQR)	36 (13)	36 (12)	39 (13)	42 (16)	39 (14)	<0.0001
Proportion of male patients %(95% CI)	47.2 (39.2–55.2)	47.4 (41.6–53.1)	54.0 (49.4–58.6)	57.8 (54.1–61.4)	52.6 (49.7–55.5)	0.001
Total viral load samples	2031	2509	4451	7741	9490	0.002
Mean viral load (sd) (log_10_)	7.56 (2.9)	7.11 (3.2)	6.14 (3.3)	5.74 (3.2)	6.75 (3.2)	<0.0001
Proportion virally suppressed %(<1000 copies/mL) (95%CI)	47.8 (36.7–67.4)	58.2 (41.4–79.6)	62.7 (45.2–84.7)	67.2 (49.0–89.9)	61.2 (43.9–83.0)	<0.0001
Proportion virally suppressed %(<400 copies/mL) (95%CI)	41.0 (29.0–48.0)	49.0 (43.0–58.0)	56.0 (48.0–64.0)	62.0 (54.0–69.0)	53.0 (45.0–60.0)	<0.001

The HIV prevalence in EMM increased from 32.3% to 36.9% from 2012 to 2016. In the same time period, the proportion of PLHIV initiating ART coverage steadily increased from 32.3% to 81.2% (Table 2).

**Table 2:** Estimated ART coverage for PLHIV (2012–2016) in antenatal care in Ekurhuleni Metropolitan Municipality.

Year	Estimated population*	Total HIV prevalence^†^	ART coverage^‡^
2012	3,219,000	32.3%	32.2%
2013	3,301,000	33.5%	56.4%
2014	3,385,000	30.2%	81.9%
2015	3,470,000	31.6%	88.0%
2016	3,559,000	36.9%	81.2%

*interpolated from Statistic South Africa mid-year estimates

^†^Estimated obtained from National Antenatal Sentinel HIV and Syphilis Survey

^‡^Estimates obtained from District Health Information System

### Viral load suppression trends (2012–2016)

Overall, in Ekurhuleni, 65.5% of the viral load test results for the threshold of <1000 copies/mL and 56.9% for a threshold of <400 copies/mL were virally suppressed. The proportion virally suppressed at <1000 copies/mL increased from 47.8% (95% CI: 36.7%-67.4%) in 2012 to 67.2% (95% CI:49.0%-89.9%) in 2015 and decreased in 2016 to 61.2% (95% CI:43.9%-83.0%). Restricting analysis to August 2016, the sensitivity analysis showed a similar pattern in 2016 where overall, the proportion of those virally suppressed reduced (S1 and S2 Figs) However, across the subdistricts, this trend was observed in the East and South subdistricts (S3 and S4 Figs). In the North subdistrict, the proportion of those virally suppressed increased in 2016. Overall, viral load suppression (<400 copies/mL) in Table 1 followed a similar pattern. The sensitivity analysis showed a similar trend of viral load suppression overall (S2 Fig). The North subdistrict showed the proportion of those virally suppressed increased from 2012 to 2014 and decreased in 2015 and increased in 2016. Sensitivity analysis at the subdistrict level in S3 and S4 Figs showed similar patterns.

### Viral load suppression in 2016

**Fig 1:**
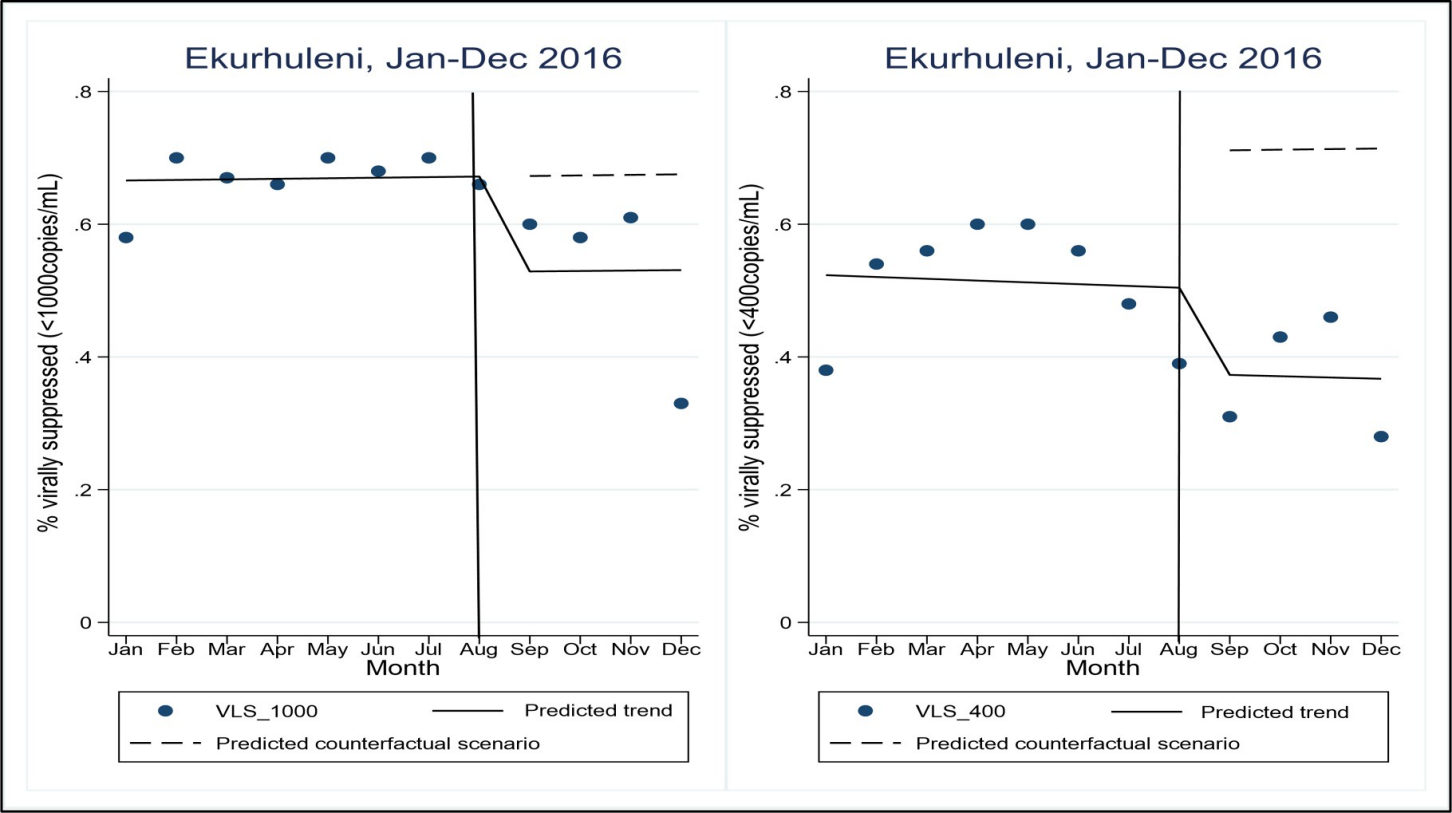
Proportions of viral load suppression rates in EMM before and after the roll-out of universal test and treat (2016).

The proportions VLS in EMM decreased immediately after the roll-out of UTT. Overall, the viral load suppression (<1000 copies/mL) ranged from 65.0% in January 2016 to 70.0% by the end of August 2016 and to 58.0% by end of December 2016. The proportion virally suppressed at <400 copies/mL was lower as shown in Fig 1 before and after UTT roll-out. Fig 1 further displays the pre-intervention trend of monthly proportions of viral load suppression (continuous line), and the counterfactual scenario (dashed line). The proportion of those virally suppressed in the post UTT phase is reduced when looking at <400 copies/ml given that most points lie below the counterfactual line which is not observed in the <1000 copies/mL trend.

### Viral load suppression post-2016

**Fig 2:**
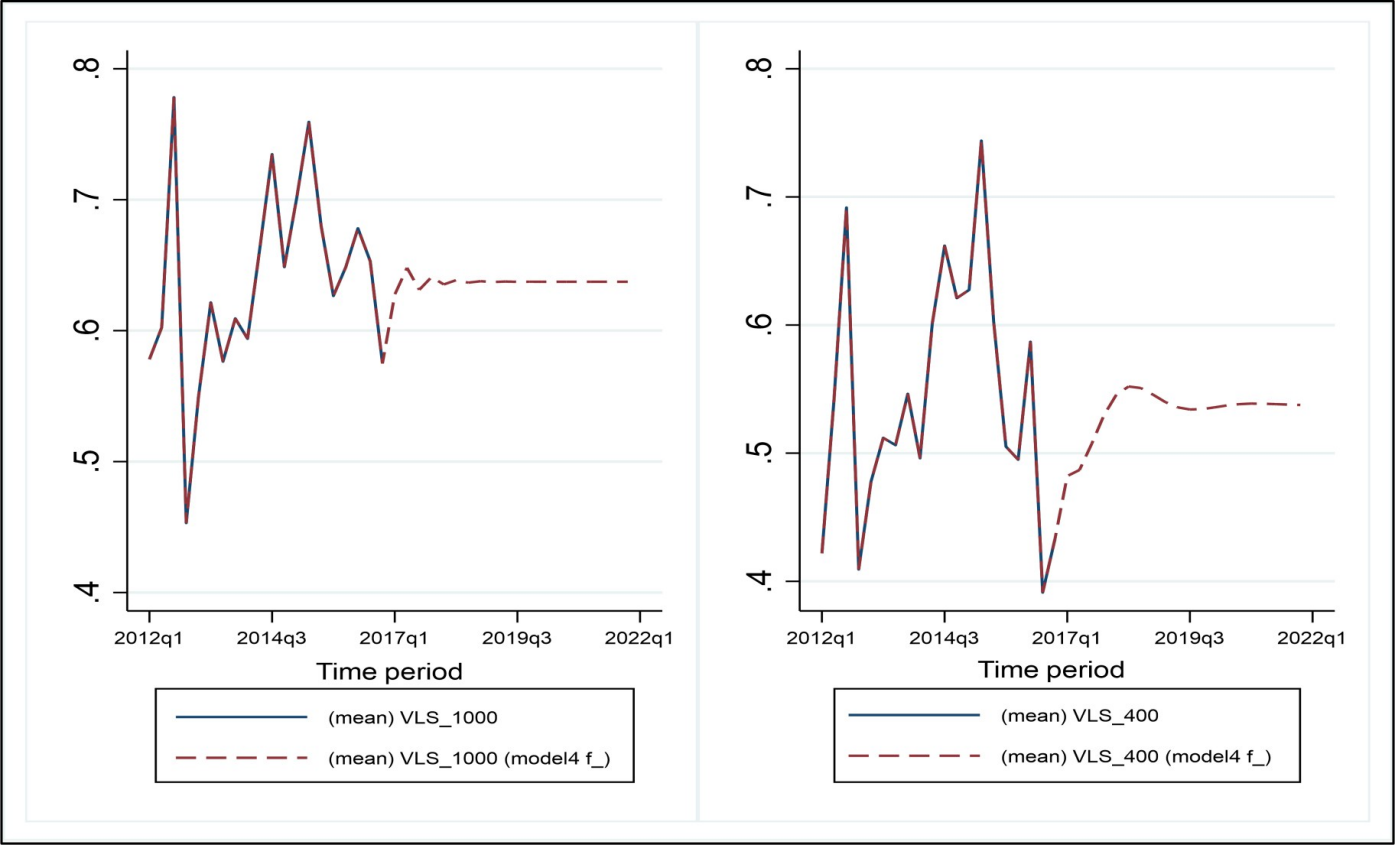
Observed (2012–2016) and predicted (2017–2021) viral load suppression rates by ARIMA modelling technique.

The predicted viral load suppression rate values matched well with the actual historical rates as seen in Fig 2. The out-of-sample forecast of viral load suppression rate for the 1000 copies/mL threshold increased in 2017 before decreasing in 2018. Conversely, the out-of-sample forecast for viral load suppression rates for the 400 copies/mL threshold increased from 2017–2019 and started to decrease beyond the second quarter of 2019.

### Spatial autocorrelation of viral load suppression

**Fig 3:**
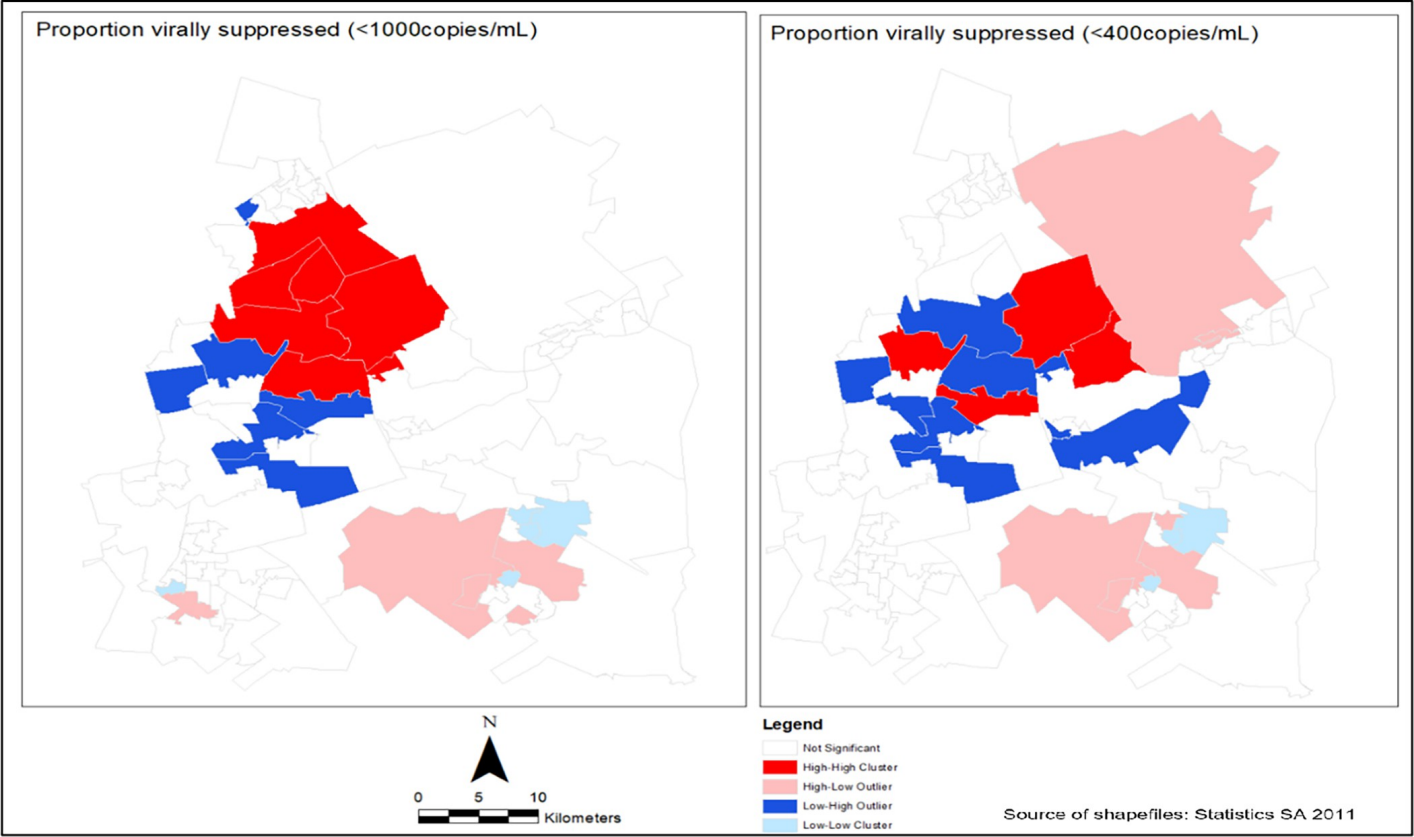
Location of clusters of high and low proportions of viral load suppression in Ekurhuleni Metropolitan Municipality (1^st^ January 2012 - 31^st^ December 2016).

To assess spatial autocorrelation of viral load suppression (<1000 copies/mL) in EMM, an estimated Moran index value of 0.51 with a statistically significant p-value (<0.0001) was calculated. Further, the estimated Moran index value of 0.10 was calculated from spatial autocorrelation of viral load suppression (<400 copies/mL) with a statistically significant p-value (<0.0001). This indicated the evidence of clusters of high or low viral load suppression in the study area. The local indicator spatial autocorrelation statistic (LISA) revealed clusters of areas with a high (blue colour) proportion of viral load suppression in the South district around Kempton Park, Alberton, Benoni and Boksburg areas. The areas with low (red colour) proportions of viral load suppression were observed in most wards in the East subdistrict in areas such as Thokoza, Brakpan and Langaville (Fig 3).

### Spatio-temporal predictors of high viral load suppression

As the proportion of PLHIV initiating on ART in a ward increased, the viral load suppression rate increased by 35% (RR: 1.345; 95% CrI 1.221–1.492). The viral load suppression rate (<1000 copies/mL) increased by 44% with an increasing proportion of female in a ward (RR: 1.442; 95%CrI: 1.056–1.962) (Table 3).

**Table 3:** Spatio-temporal predictors of higher viral load suppression in Ekurhuleni (2012–2016).

	<1000 cp/mL		<400 cp/mL	
Predictor	Beta co-efficient Mean (95%CrI)	Exponentiated co-efficient) RR (95%CrI)	Beta co-efficient Mean (95%CrI)	Exponentiated (co-efficient) RR (95%CrI)
Population density	0.012 (0.001–0.024)	1.008 (0.993–1.023)	0.001 (-0.009–0.012)	1.001 (0.991–1.012)
% Diagnosed with TB	-0.006 (-0.017–0.005)	0.994 (0.983–1.004)	-0.006 (-0.016–0.004)	0.994 (0.983–1.005)
**% Initiated on ART**	**0.300 (0.200–0.400)**	**1.345 (1.221–1.492)**	**0.200 (0.100–0.300)**	**1.221 (1.052–1.350)**
**% Female**	**0.335 (0.085–0.585)**	**1.442 (1.056–1.961)**	0.302 (-0.024–0.626)	1.352 (0.976–1.870)
% no literacy	-0.325 (-1.559–0.907)	0.563 (0.104–3.027)	0.527 (-1.163–2.198)	1.693 (0.312–9.003)
% no income	1.066 (-0.852–2.983)	3.835 (0.440–33.630)	0.297 (-1.873–2.452)	1.346 (0.154–11.614)
Constant	0.166 (-0.013–0.346)	1.194 (0.964–1.486)	0.186 (-0.043–0.420)	1.204 (0.958–1.523)

### Temporal changes in parameters and association with viral load suppression

**Fig 4:**
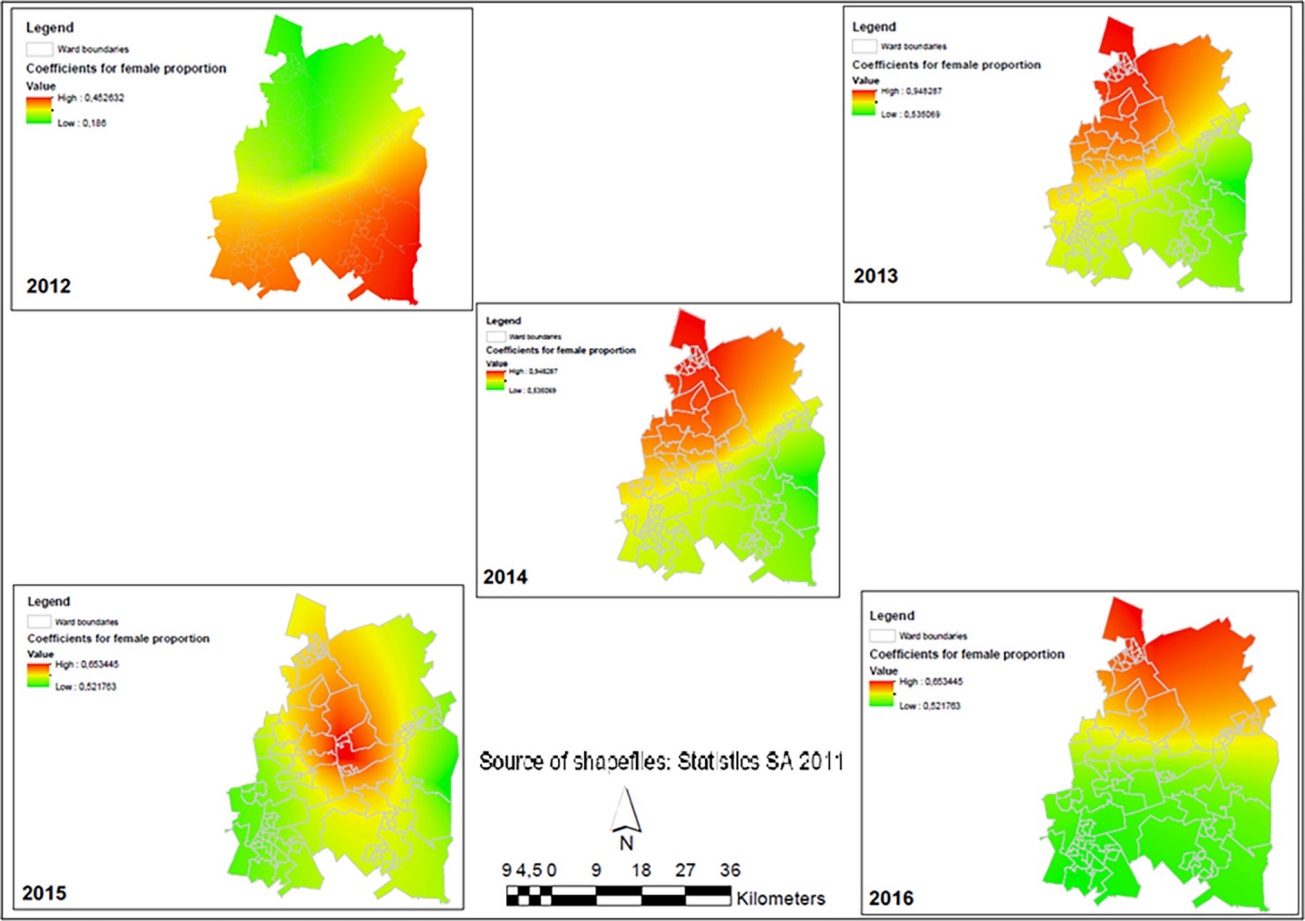
Spatial-temporal changes showing the relationship between the proportion of female population and viral load suppression (<1000 cp/mL) rates in Ekurhuleni Metropolitan Municipality (2012–2016).

Female population and ART initiation are two important predictors of viral load suppression in Ekurhuleni but their relationship was stronger in some wards and weaker in others. The patterns of these two explanatory variables were randomly distributed in all years except 2014 (Moran index = 0.077, p-value = 0.040) whose distribution was clustered. However, after adjusting for spatial effects, the pattern showed a random distribution. The relationship between the proportion of female population and high viral load suppression in Ekurhuleni changed between 2012 and 2016 (Table 4). In 2013 (β = 0.776; p-value <0.001), 2014 (β = 0.523; p-value 0.009), and 2015 (β = 0.561; p-value 0.015) this relationship was statistically significant. Spatially, the red areas were those wards in Ekurhuleni where an increase in the proportion of women was a strong predictor of high VLS. The green areas were wards where an increasing female population was not a strong predictor (Fig 4).

**Table 4:** Spatio-temporal changes of parameters associated with high viral load suppression.

	2012	2013	2014	2015	2016
	Estimate	Estimate	Estimate	Estimate	Estimate
	*P-Value*	*P-Value*	*P-Value*	*P-Value*	*P-Value*
Ordinary least squares regression results					
Intercept	0.034	0.023	0.006	0.032	0.031
	*0*.*240*	*0*.*219*	*0*.*776*	*0*.*118*	*0*.*091*
Prop Female	0.374	0.776	0.523	0.561	0.254
	*0*.*053*	*<0*.001*	*0*.009*	*0*.015*	*0*.*092*
Prop ART initiation	0.713	0.258	0.424	0.409	0.530
	*0*.004*	*0*.032*	*0*.001*	*0*.002*	*<0*.001*
Adjusted R^2^	0.253	0.434	0.527	0.601	0.606
Koenker statistic†	17.117	9.151	7.225	8.429	10.532
	*<0*.001*	*0*.010*	*0*.027*	*0*.016*	*0*.005*
Moran index	-0.047	0.005	0.077	0.042	0.042
	*0*.*415*	*0*.*701*	*0*.040*	*0*.*209*	*0*.*204*
Geographically weighted regression results					
Adjusted R^2^	0.294	0.482	0.541	0.596	0.612
Moran index	-0.050	-0.017	0.034	0.019	0.007
	*0*.*367*	*0*.*910*	*0*.*289*	*0*.*467*	*0*.*662*

†- a statistically significant p-value indicates relationship between the explanatory variables and viral load suppression is non-stationary

**Fig 5:**
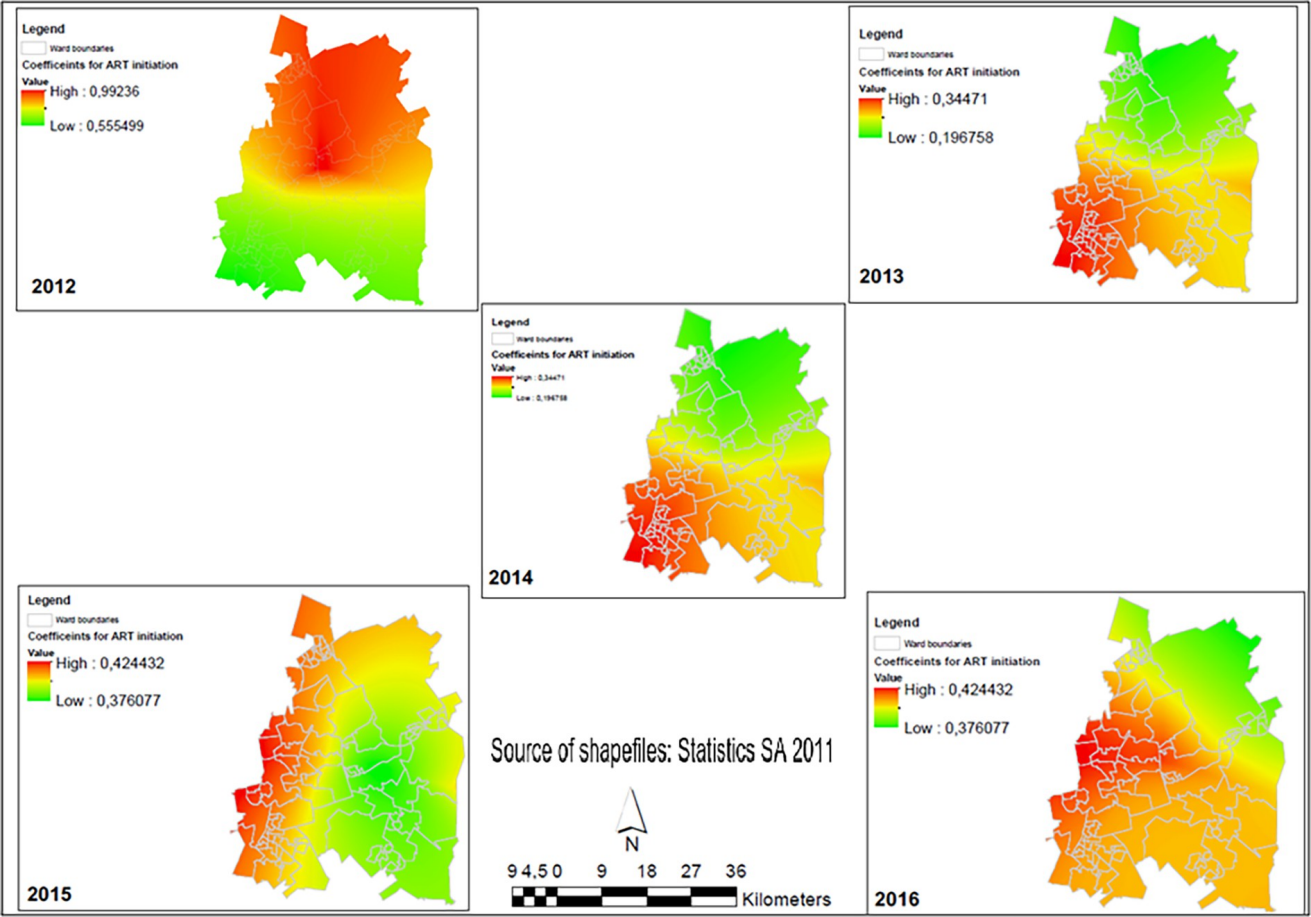
Spatial-temporal changes showing the relationship between the proportion of ART initiation and viral load suppression (<1000 cp/mL) rates in Ekurhuleni Metropolitan Municipality (2012–2016).

Similarly, the relationship between the proportion ART initiation and high viral load suppression in Ekurhuleni changed between 2012 and 2016 (Table 4). The relationship was statistically significant across all the years, namely: In 2012 (*β* = 0.713; p-value <0.004), 2013 (*β* = 0.258; p-value 0.032), 2014 (*β* = 0.424; p-value 0.001), 2015 (*β* = 0.409; p-value 0.002), and 2016 (*β* = 0.530; p-value <0.001). Spatial patterns reveal that the red areas were those wards in Ekurhuleni where an increase in the proportion of PLHIV initiating on ART was a strong predictor of high viral load suppression. The green areas were those wards where an increasing ART population was not a strong predictor (Fig 5).

## Discussion

These findings show the presence of spatial and temporal heterogeneity in viral load suppression in Ekurhuleni using routinely collected laboratory data. These further provide valuable insights into the HIV programmes in the study area by identifying wards needing additional support for linkage to and retention in care. Although the proportion of virally suppressed PLHIV steadily increased from 2012 to 2015, the spatial maps revealed ward-level differences and evidence that high and low levels of viral load suppression rates were clustered geographically across space and time. High viral load suppression rates were positively correlated to increasing ART coverage and female population. However, this finding did not apply to the study area over the five years. When the threshold of <400 copies/mL was applied, the viral load suppression decreased by at most 15%. This highlights the gaps in monitoring, treatment, and availability of more effective ART. In South Africa, studies have shown that at an individual level, being female is a predictor of viral load suppression. A multi-centre study in KwaZulu Natal, Johannesburg and Cape Town showed that adult women were 26% more likely to report greater viral load suppression rates [15]. Our findings, however, showed that an increasing female population in Ekurhuleni was likely to reduce the viral load suppression rate at a ward level. Another study on pregnant women using the 2017 national antenatal survey, highlighted the failure to achieve viral load suppression by the third trimester [22]. Pregnancy is a known predictor of poor viral load suppression [23]. Delayed entry into antenatal care, delayed initiation, poor adherence, and infrequent clinic visits during the 3^rd^ trimester were the main reasons cited for a viral load >50 copies/mL [22, 24]. Although this study was not specific to Ekurhuleni, it underscores the importance of improving early antenatal care and ART initiation among pregnant women and improving HIV care to women living with HIV. Findings also showed that increasing the proportion of women was a strong predictor of VLS in some wards not in others. Focused strategies in retaining women in care are needed.

Understanding the variations in viral load suppression across space and time can guide interventions to improve programmes monitoring retention in care, which may lead to reduces HIV transmission and HIV incidence in EMM. Controlling the HIV epidemic in the age of universal testing and treatment requires targeting individuals in the right areas. Areas with high and low-performing healthcare facilities at a ward level should be identified. In Ekurhuleni, the currently reported ART coverage is 72%. All facilities reporting less than 95% VLS should expand community-level HIV programmes to improve ART coverage. To reach the 95% target, efforts in reaching males should be made in addition to focusing on women who mostly access clinics. Facilities should find the missing men in communities who disengage from health seeking [25]. Most antenatal studies in South Africa have been used to evaluate viral load suppression in women [22, 26, 27]. Across the studies, adolescent girls, and young women (AGYW) have reported the lowest viral suppression rates [22, 26, 27]. Findings further suggest that this may be due to delayed ART initiation mostly occurring after pregnancy due to late testing, shock, need for counselling, acceptance of positive serostatus, and fear of wanting to be seen taking ART by peers and family [26, 28]. Viral load suppression is reported in married women, suggesting a presence of social support structures that encourage disclosure of HIV-positive status to partners facilitating adherence to ART [22, 27, 28]. These previous findings highlight the need for targeted approaches to reach different key groups of female individuals, specifically those in the younger age groups [22, 26–28] to improve viral load suppression rates.

In our study, we found that in the highest quartile, 86% of PLHIV achieved viral suppression and whereas for those in the lowest quartile, only 35% were virally suppressed. To ensure that viral load suppression improves in Ekurhuleni, targeting wards with lower viral load suppression is necessary. Improving the performance along the care cascade is one key step toward HIV elimination. At the ward level, facilities could improve their performance by improving the indicators in the care continuum. For instance, adherence counselling before ART initiation needs examining. Understanding the comprehension of counselling messages by patients’ needs assessment. Our findings show that more than one viral load test was conducted for each patient per year suggesting alignment with national guidelines. The number of viral load samples tested increased over the five years suggesting increased retention in care and viral load suppression. At an individual level, one study in South Africa showed that viral load suppression (<400 copies/mL) was associated with having a viral load measurement two months within the scheduled date in the facility [15]. Although our study did not set out to investigate this association, the findings are necessary to improve viral load monitoring at the facility or ward level.

Our findings show that the proportion of viral load suppression increased between 2012 and 2015 but dropped in 2016. In 2016, universal tests and treatments were introduced for all people testing HIV positive. This may have led to an increase in the number of people presenting at healthcare facilities for HIV care, increasing the denominator thus decreasing the rate of viral load suppression as observed in 2016. In addition, the large number of patients who initiated immediate treatment may have stretched the existing constrained resources and reduced efforts on counselling and follow-up. However, a recent modelling study assessing district-level targets for 90-90-90 in 2018, showed that viral load suppression in Ekurhuleni was still low at 41.3% [29]. This highlights the need to improve viral load monitoring as these recent estimates predict viral load suppression to 86% by 2030 [29]. In the era of UTT, more nuanced approaches are required to identify at a clinic and laboratory level, patients at risk for poor adherence. A clinic prediction score developed from a previous study using routinely collected data showed better performance over standard adherence measures in correctly identifying patients likely to be poor adherers at 6 months post ART initiation [30]. These patients will subsequently require individualized treatment protocols to prevent virologic failure as has been observed in some developed countries [31, 32].

Our findings showed a strong relationship between increasing the proportion initiating ART and viral load suppression rates at the ward level which confirmed previous findings in other settings which showed that widely increasing ART initiation leads to improved viral load suppression rates [33]. Further, the heterogeneity of this strong prediction was seen across the ward over the study period. These findings show the heterogeneity of HIV program in Ekurhuleni which needs focused improvement. Services such as same-day initiations and differentiated delivery of care to are needed [34, 35] to increase the proportion of those on treatment and interruptions in ART initiation and retention [29]. Interruptions are likely to introduce resistance and the introduction of the integrase strand-transfer inhibitor dolutegravir, may increase overall virological suppression rates [36]. However, more needs to be done to alleviate concerns regarding the management of viremia.

Our analysis has identified geographic disparities and recommends strengthening targeted adherence at the ward level by improving counselling and management of the patients to address ward inequalities. Continued exploration of the interaction of population dynamics and programmatic interventions such as viral load testing access or coverage and enhanced adherence for individuals on treatment and those with virologic failure are needed [37]. For the poor-performing wards, strengthening tracing programmes which track patients who default or those living in adjacent wards requires collaboration between wards. Facilities serving communities in Thokoza, Langaville and Brakpan need to intensify these efforts to ensure that PLHIV is retained in care to achieve viral load suppression.

This analysis makes several contributions to the literature. First, we used existing routinely collected laboratory data to monitor viral load in Ekurhuleni and used other readily available databases. Secondly, we used Bayesian analytical techniques which incorporated longitudinal design to assess the predictors of viral load suppression over time rather than cross-sectional estimates [38]. Thirdly, this study provided a robust approach to spatial analysis whereby geospatial data was used to evaluate underlying patterns of viral load suppression within a small area using local surveillance data. Our analysis had some weaknesses, and the findings should be interpreted with caution. First, some variation in our ward-level viral load suppression measure may reflect random fluctuations. However, by averaging over the five years of data, we attained a measure with high reliability, indicating evidence of a persistent ward effect. Secondly, we used laboratory data through December 2016 three months into UTT implementation and did not have enough data post-implementation for comparison. Nevertheless, using forecasting, we predicted the viral load suppression in Ekurhuleni between 2017 and 2021. In addition, there were potential missing laboratory data that may have originated from lost to follow-up. Thirdly, we used data from patients in HIV care who may frequently test for viral load suppression due to poor adherence and may have missed including data from those who do not frequently attend clinic visits, those who attend clinics in NGO settings or conduct pharmacy pickups. Additionally, the data we report does not discriminate between non-key and key populations such as sex workers, a population with high HIV prevalence rates. Fourthly, the absence of health facility characteristics from this analysis limited accounting for the differences across the different health facilities. Adding other factors including related information from the health system level and HIV drug resistance would improve the predictive value of the model and improve forecast accuracy. Lastly, our findings may not be generalizable to other settings but may be representative of other high-priority districts.

### Conclusions

Wards with facilities that successfully monitor PLHIV for viral suppression are likely to see a reduction in HIV prevalence in the communities they serve. Wards with facilities reporting poorer performance will experience persistent transmission and other poor outcomes. Addressing gaps that target adherence, and retention in care for men is critical to achieving optimal viral load suppression in Ekurhuleni. Measuring differences in viral load across wards and visualizing their variations is a critical step toward viral load suppression.

Ekurhuleni’s public health strategy should increase the intensive implementation of retention programmes and wider efforts around HIV viral suppression in these geographic areas to achieve the goals of the 90-90-90 plan.

## Supporting information

S1 Data (XLSX)

S1 Fig Overall proportion of viral load suppression (<1000 copies/mL) Ekurhuleni Metropolitan Municipality (1^st^ January 2012 - 31^st^ August 2016). (TIF)

S2 Fig Proportion of viral load suppression (<1000 copies/mL) by sub-district in Ekurhuleni Metropolitan Municipality (1^st^ January 2012 - 31^st^ August 2016). (TIF)

S3 Fig Overall proportion of viral load suppression (<400 copies/mL) by sub-district in Ekurhuleni Metropolitan Municipality (1^st^ January 2012 - 31^st^ August 2016). (TIF)

S4 Fig Proportion of viral load suppression (<400 copies/mL) by sub-district in Ekurhuleni Metropolitan Municipality (1^st^ January 2012 - 31^st^ August 2016). (TIF)
