# Supplementary figures and images for: Small area analysis of HIV viral load suppression patterns in a high priority district (2012–2016), South Africa
Source: PLOS Glob Public Health. 2023 Mar 31;3(3):e0001728. doi: 10.1371/journal.pgph.0001728 (PMC10065273; doi:10.1371/journal.pgph.0001728)

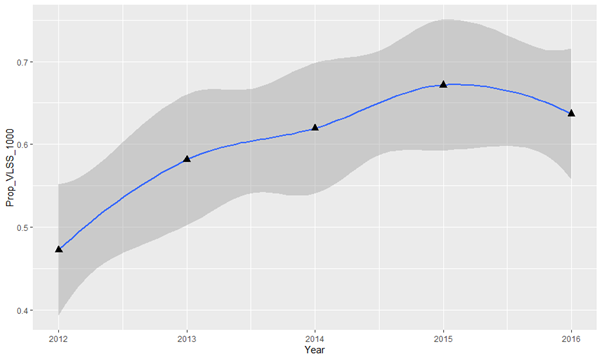

Supplement: S1 Fig — (TIF) [file pgph.0001728.s002.tif]

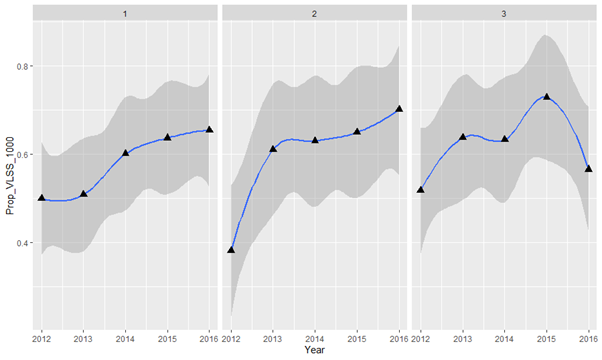

Supplement: S2 Fig — (TIF) [file pgph.0001728.s003.tif]

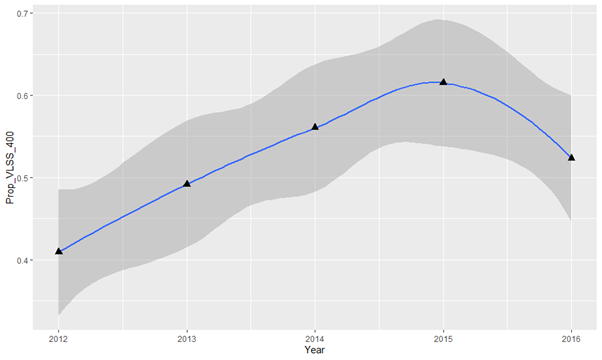

Supplement: S3 Fig — (TIF) [file pgph.0001728.s004.tif]

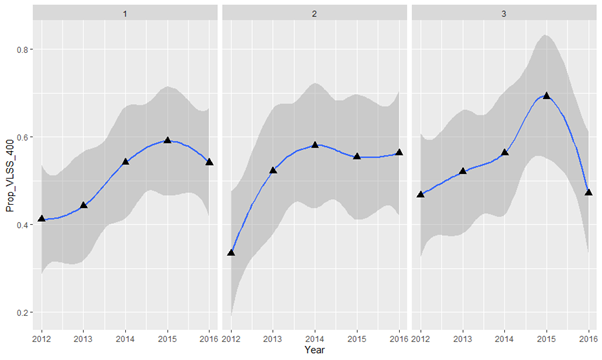

Supplement: S4 Fig — (TIF) [file pgph.0001728.s005.tif]
